# CD4^+^ T Cells Alter the Stromal Microenvironment and Repress Medullary Erythropoiesis in Murine Visceral Leishmaniasis

**DOI:** 10.3389/fimmu.2018.02958

**Published:** 2018-12-18

**Authors:** Olivier Preham, Flaviane A. Pinho, Ana Isabel Pinto, Gulab Fatima Rani, Najmeeyah Brown, Ian S. Hitchcock, Hiro Goto, Paul M. Kaye

**Affiliations:** ^1^Centre for Immunology and Infection, Hull York Medical School and Department of Biology, University of York, York, United Kingdom; ^2^Laboratório de Soroepidemiologia e Imunobiologia, Faculdade de Medicina, Instituto de Medicina Tropical de São Paulo, Universidade de São Paulo, São Paulo, Brazil

**Keywords:** erythropoiesis, stromal cells, macrophages, bone marrow, leishmaniasis

## Abstract

Human visceral leishmaniasis, a parasitic disease of major public health importance in developing countries, is characterized by variable degrees of severity of anemia, but the mechanisms underlying this change in peripheral blood have not been thoroughly explored. Here, we used an experimental model of visceral leishmaniasis in C57BL/6 mice to explore the basis of anemia following infection with *Leishmania dono*v*ani*. 28 days post-infection, mice showed bone marrow dyserythropoiesis by myelogram, with a reduction of TER119^+^ CD71^−/+^ erythroblasts. Reduction of medullary erythropoiesis coincided with loss of CD169^high^ bone marrow stromal macrophages and a reduction of CXCL12-expressing stromal cells. Although the spleen is a site of extramedullary erythropoiesis and erythrophagocytosis, splenectomy did not impact the extent of anemia or affect the repression of medullary hematopoiesis that was observed in infected mice. In contrast, these changes in bone marrow erythropoiesis were not evident in B6.*Rag2*^−/−^ mice, but could be fully reconstituted by adoptive transfer of IFNγ-producing but not IFNγ-deficient CD4^+^ T cells, mimicking the expansion of IFNγ-producing CD4^+^ T cells that occurs during infection in wild type mice. Collectively, these data indicate that anemia during experimental murine visceral leishmaniasis can be driven by defects associated with the bone marrow erythropoietic niche, and that this represents a further example of CD4^+^ T cell-mediated immunopathology affecting hematopoietic competence.

## Introduction

The bone marrow (BM) is the main site of hematopoiesis in adult mammals and occurs within the cavities of long bones. Hematopoiesis is a complex process through which hematopoietic stem cells (HSCs) proliferate and differentiate into mature blood cells and is largely restricted to specific microenvironments or “niches” that are comprised of a variety of non-hematopoietic stromal cells and secreted factors. The stromal cell-derived chemokine CXCL12 and its receptor CXCR4 are responsible for the retention of HSCs in the BM. Disruption of the CXCL12-CXCR4 axis, or depletion of CXCL12-abundant reticular (CAR) cells, mobilizes HSCs in the peripheral blood (1). A wide spectrum of diseases impact on hematopoiesis in general and on erythropoiesis in particular by altering these niches, including myeloproliferative neoplasms and infectious diseases (2). For example, *Escherichia coli* and *Anaplasma phagocytophilum* infections in murine models has been shown to induce CXCL12 down-regulation in the BM and subsequent HSC mobilization (3, 4). The development of anemia is often complex and multifactorial, as evidenced by experimental studies in infectious disease models and often reflects a balance between erythropoiesis and erythrocyte clearance. For example, in *Trypanosoma brucei* infection, anemia is in part caused by nitric oxide (NO) production, and pro-inflammatory cytokines, such as IFNγ and TNF positively correlate with anemia severity (5). In contrast, direct lysis of RBC is seen during acute malaria (6). CD169^+^ BM stromal macrophages are also an essential component of the niche for erythropoiesis (7) as well as important regulators of stromal cells within the HSC niche (8, 9), but less is known about how their function is impacted during infection, or in relation to the development of anemia.

Hematological disturbances are a hallmark of human and canine visceral leishmaniasis (VL) (10, 11), caused by infection with the protozoan parasites *Leishmania donovani* or *L. infantum*. Differing degrees of cytopenia are associated with disease stage, and as risk factors for VL-related death (12, 13). VL often results in pancytopenia (14–16) and may sometimes be misdiagnosed as another hematological disorder, such as myelodysplastic syndrome (17). Various mechanisms have been proposed to underpin the development of VL-associated pancytopenia, including auto-immune destruction of erythrocytes, platelets and leukocytes, or BM failure (18). Anemia has been attributed to aberrant sialoglycosylation of red blood cells (19), altered recognition of band 3 subsequent to oxidative stress (20) or enhanced macrophage-mediated erythrophagocytosis (21).

While the immune response and hematological consequences of VL have been extensively studied, far less is known about the regulation of hematopoiesis *per se* during disease, in part due to the ethical challenges involved in studying this in humans. Hematopoiesis has been examined in a hamster model of VL (22), with the finding that *L. donovani* infection induces apoptosis in erythropoietic progenitors in the BM. However, lack of tools for dissecting the hamster immune and hematopoietic microenvironment poses challenges in exploiting this model. Although the mouse model of VL is not lethal, it has been extensively studied to provide more mechanistic data on immunity and immunopathology (23, 24). However, this model has to date been poorly utilized in the study of hematological dysfunction. Cotterell et al. demonstrated that chronic VL in BALB/c mice results in an increase of hematopoietic progenitors in the spleen and the BM (25), and that BM stromal macrophage-derived cells may become more supportive of myelopoiesis after infection with *L. donovani in vitro*, due to increased secretion of GM-CSF and TNF (26). More recently, alterations in the HSC compartment have been described that might contribute both to ongoing VL-associated immunosuppression (27) and to long term hematopoietic competence (28).

Here, we have focused on exploring the mechanisms underpinning anemia in C57BL/6 mice infected with *L. donovani*. We show that infected mice develop BM dyserythropoiesis, evidenced both by myelogram and by a reduction of medullar TER119^+^ CD71^−/+^ erythroblasts. Reduction of medullary erythropoiesis coincided with loss of CD169^high^ stromal macrophages and a reduction of CXCL12-expressing stromal cells. We demonstrate, through the use of immunodeficient B6.*Rag2*^−/−^ mice and adoptive cell transfer, that all of these events strictly require the presence of CD4^+^ T cells expressing IFNγ. Hence, we propose that repression of medullary erythropoiesis is added to the catalog of immunopathological sequelae associated with *Leishmania donovani* infection.

## Material and Methods

### Ethics Statement

All animal care and experimental procedures were performed under UK Home Office License (Ref # PPL 60/4377) and with approval from the Animal Welfare and Ethical Review Board of the Department of Biology, University of York.

### Mice

C57BL/6, B6.*Rag2*^−/−^, B6.*Cxcl12*^tm2.1Sjm/J^ mice (Jackson Laboratories) and B6.hCD2-DsRed mice were bred at the University of York. IFNγ-KO (B6.129S7-*Ifng*tm1Ts/J, stock no. 002287) mice were obtained from the Jackson Laboratory. All mice were maintained under specific pathogen-free conditions (FELASA 67M standard). As appropriate, mice were micro-chipped, randomly allocated to groups and infected intravenously with 2–3 × 10^7^ *L. donovani* (LV9) amastigotes isolated from the spleen of infected B6.*Rag2*^−/−^ mice. Mice were splenectomized (Sp_x_) or sham-operated by a commercial supplier (Charles River UK), and were allowed to recover for 3 weeks before being infected. As required, 6 × 10^5^ sort-purified splenic CD45^+^CD4^+^CD3^+^CD8^−^B220^−^TCRγδ^−^CD49b^−^ cells derived from wild type or IFNγ-KO mice were transplanted into B6.*Rag2*^−/−^.CD45.1Cg recipient mice 24 h prior to infection.

Unless stated otherwise, experimental mice were killed by cervical dislocation 4 weeks after infection.

### Blood Analysis

Blood was collected from terminally anesthetized mice by cardiac puncture in syringes coated with Citrate-dextrose and transferred into a EDTA-coated Vacutainer®. Blood analysis was performed with a Hemavet 950FS (Drew Scientific).

### Bone Marrow Myelogram

BM samples were obtained by aspiration biopsy from the iliac crest using a 24 G needle attached to a 5 mL disposable plastic syringe with 10% EDTA and smears were stained with May–Grünwald Giemsa. Samples were then re-coded for blind analysis. A differential count of 500 cells was made in BM smears to calculate: myeloid: erythroid (M:E) ratio, the myeloid maturation ratio, the erythroid maturation ratio, myeloid precursor cells (myeloblasts + promyelocyte + myelocyte), percentages of myeloid mature cells (metamyelocyte + band neutrophils + segmented neutrophils), erythroid precursor cells (CD71^+^TER119^lo^ proerythroblasts + CD71^−/+^TER119^high^ basophil erythroblasts), erythroid mature cells (polychromatic erythrocyte + orthochromatic erythrocytes; equivalent to CD71^−/+^ TER119^high^), monocytes, macrophages, plasma cells, and megakaryocytes according to Yang et al. (29). The dysplasic features were also analyzed in the myeloid and erythroid series and in megakaryocytes.

### Immunohistochemistry

Femurs were isolated and cleaned to remove excessive tissue then fixed overnight at 4°C in periodate-lysine-paraformaldehyde fixative [10 mM sodium periodate dissolved in three parts 0.1 M lysine-HCl 0.1 M Na_2_HPO_4_ and one part 20% (w/) paraformaldehyde] and decalcified for 3 days at 4°C with slow agitation in 10% EDTA, 0.1 M Tris, pH6.95. Bones were transferred in 30% sucrose in PBS for a final overnight incubation at 4°C. Spleen and bones were embedded in Optimal Cutting Temperature (OCT™) compound (Tissue-Tek) in Cryomolds® (Tissue-Tek) and snap-frozen on dry ice. Spleen and femoral 5 μm-sections were cut using a CM1900 cryostat (Leica Microsystems) onto Polysines® slides (Thermo Fisher). Spleen section were fixed in ice-cold acetone for 10 min on the day of staining. Sections were blocked in staining buffer [PBS, 0.05% (w/v) BSA, 5% goat serum] for 1 h at RT. Excess buffer was removed and slides stained with fluorochrome-labeled TER119, F4/80, CD71 or isotype controls (eBioscience) in staining buffer for 1 h at RT or overnight at 4°C. Slides were washes three times for 5 min in washing buffer (PBS 0.05% (w/v) BSA) and counterstained with DAPI. Section were mounted in ProLong® Gold antifade reagent (Life Technologies) and sealed before imaging. Confocal images were obtained using LSM780 or LSM710 systems (Carl Zeiss) and analyzed using Zen software (Carl Zeiss). Samples were assessed blind to treatment group.

### Flow Cytometry

Spleen cells were dissociated using a 70 μm cell strainer. Femurs were cut at both ends to expose the bone cavity and the BM was flushed with PBS 1% FCS (flow cytometry buffer) using a 25-gauge needle through a 70 μm cell strainer. Single cell suspensions were washed (5 min at 300 g) and red blood cells were lysed with ACK buffer (5 min at RT). Nucleated cells were subsequently counted using a Vi Cell XR Cell Counter (Beckman Coulter). Cell suspensions were incubated in FcBlock (mouse CD16/32 purified antibody, clone 93) prior to staining with antibodies specific for CD71 (clone R17217), TER119 (clone TER-119), and CD45 (clone 30-F11) or with F4/80 (clone BM8), Ly-6G (clone Gr-1), CD115 (clone AFS98), and CD169 (clone SER-4). For T cell characterization, cells were labeled with in optimized concentration of flurochrome-labeled CD45, CD4 (clone RM4-5 or GK1.5), CD8 (clone 53-7.7), TCRγδ (clone GL-3), B220 (CD45R; clone RA3-6B2), CD49d (clone DX-5), and CD3 (clone 145-2C11) antibodies diluted in 1 × PBS 1% FCS and left at 4°C for 30 min in the dark. Cells were washed and analyzed on a Cyan flow cytometer (Beckman Coulter).

### Statistical Analysis

Data were analyzed using GraphPad Prism 5.0 (Prism Software, Irvine, CA, USA). When comparing two groups, Student's *t*-test or Mann-Whitney test was used according to the data distribution. Welch's correction was applied for the Student's *t*-test in cases of unequal variances between the two groups. For multiple comparison, one-way ANOVA or Kruskal-Wallis tests were used according to the data distribution followed by Turkey's or Dunn's multiple comparison tests, respectively. Downstream analyses were performed blind to treatment group.

## Results

C57BL/6 mice were infected with *L. donovani* amastigotes by the intravenous route and blood parameters were measured over time. Data from naïve mice (*n* = 14) were used to calculate the reference interval, or normal range, for each parameter in the complete blood count. Anemia was first evident at week 4 post-infection (Table 1, Table S1), a time that also represents the approximate peak of infection in spleen and bone marrow (28). The mean red blood cell (RBC) count per μl of blood was 19% lower in infected mice compared to their naïve counterparts. 70% of infected mice had RBC counts below the normal range. Similarly, the mean hemoglobin (Hb) content in the blood of infected mice was decreased by ~15% in infected mice and ~30% of infected mice had Hb levels below the reference interval. The average volume of erythrocytes was unchanged, with a mean corpuscular volume (MCV) of 51 femtoliter (fl) in both groups but 3/13 infected mice (23%) had developed a macrocytic anemia. Although the overall hemoglobin concentration was reduced, all individual mice had mean corpuscular hemoglobin (MCH) values within the normal range. Blood film examination indicated the presence of aberrant red cell morphology with aniso-poikilocytosis, polychromasia, acanthocytes and nucleated red cells (Figure S1). No significant change in circulating lymphocytes, granulocytes or monocytes was measured between naïve and infected mice, except for a single infected mouse that presented with both lymphopenia and eosinophilia. Thrombocytopenia was evident. These results all point toward development of a normochromic anemia coupled with thrombocytopenia as the most common hematological consequences of *L. donovani* in C57BL/6 mice.

**Table 1:** Hematological characteristics of C57BL/6 mice infected for 28 days with *L. donovani*.

	**Naive**	**Infected**
WBC (×10^3^/ul)	6.803 ± 0.864	5.758 ± 0.659
NE (×10^3^/ul)	1.671 ± 0.309	1.108 ± 0.128
LY (×10^3^/ul)	4.486 ± 0.455	4.072 ± 0.626
MO (×10^3^/ul)	0.296 ± 0.072	0.230 ± 0.017
EO (×10^3^/ul)	0.259 ± 0.077	0.108 ± 0.058
BA (×10^3^/ul)	0.077 ± 0.026	0.013 ± 0.003
RBC (×10^6^/ul)	8.110 ± 0.143	6.572 ± 0.241^***^
HB (g/dl)	9.593 ± 0.213	8.169 ± 0.219^***^
HCT (%)	41.860 ± 0.900	34.020 ± 1.091^***^
MCV (fl)	51.610 ± 0.577	51.990 ± 1.035
MCH (pg)	11.860 ± 0.227	12.520 ± 0.198^*^
MCHC (g/dl)	23.040 ± 0.663	24.130 ± 0.509
PLT (×10^3^/ul)	583.000 ± 45.680	281.500 ± 26.39^***^
MPV (fl)	4.293 ± 0.143	5.354 ± 0.084^***^

Bold values are significant:

* p < 0.05;

*** *p < 0.0001*.

### Compensatory Extra-Medullary Erythropoiesis Occurs in the Spleen but Medullary Erythropoiesis Is Repressed During EVL

**Figure 1:**
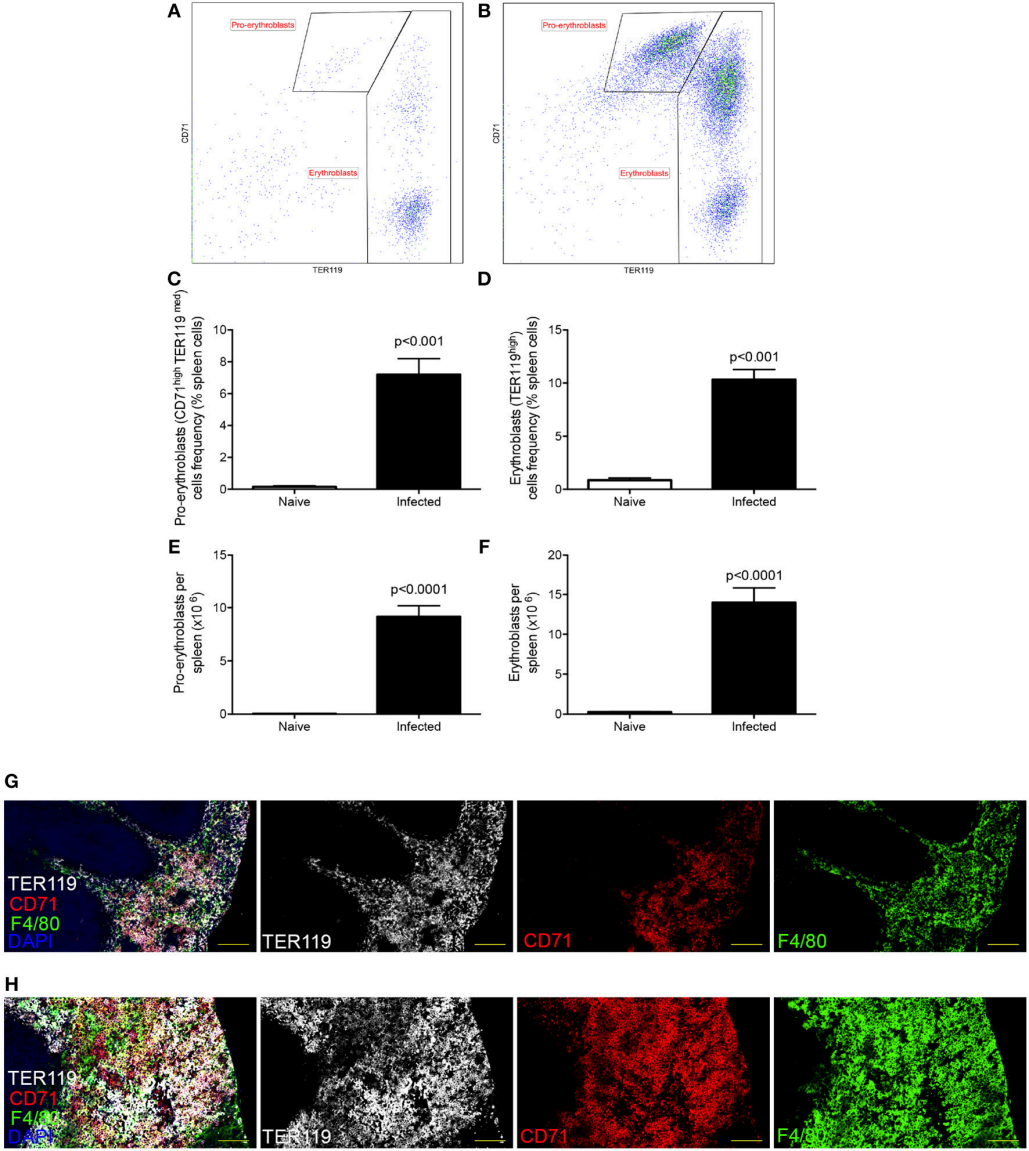
*L. donovani* infection induces extramedullary erythropoiesis in the spleen. **(A,B)** Gating strategy for identification of pro-erythroblasts (CD45^−^CD71^high^TER119^low^) and erythroblasts (CD45^−^CD71^high/low^TER119^high^) in the spleens of naïve **(A)** and infected **(B)** mice. Plots are gated on CD45^−^ live cells and equal number of live cells. **(C)** Frequency of pro-erythroblasts in the spleen. **(D)** Frequency of erythroblasts in the spleen. **(E)** Absolute number of pro-erythroblasts per spleen. **(F)** Absolute number of erythroblasts per spleen. Absolute numbers were calculated by multiplying the cell frequencies by the total numbers of cells per spleen. **(G,H)** Representative histology of spleens from control **(G)** and infected **(H)** mice. Sections were stained for F4/80 (green), TER119 (white), CD71 (red), and counterstained with DAPI (Blue). F4/80 demarcates the red pulp. All mice were infected for 28 days. Data represent mean ± SEM (unpaired *t*-test with Welch's correction; *n* = 8 mice per group from two independent experiments).

Decrease in hematocrit can be caused by reduced numbers of circulating erythrocytes, by impairment of erythropoiesis or by peripheral destruction of RBC. Others have previously reported erythrophagocytosis occurring in the spleen during experimental VL (21), associated with splenomegaly. However, the spleen is also well-known as a site with a propensity for extramedullary hematopoiesis. We confirmed that splenomegaly was associated with extra-medullary erythropoiesis (Figure 1), as determined by an increased frequency (Figures 1C,D) and absolute number (Figures 1E,F) of CD45^−^CD71^high^TER119^low^ pro-erythroblasts and CD45^−^CD71^high/low^TER119^high^ erythroblasts (30). CD71^+^TER119^+^ cells localized predominantly within the enlarged red pulp (Figure 1G). Hence, during experimental VL, splenomegaly provides both an environment in which splenic clearance of RBCs can occur (21), as well as an environment conducive to enhanced compensatory erythropoiesis.

**Figure 2:**
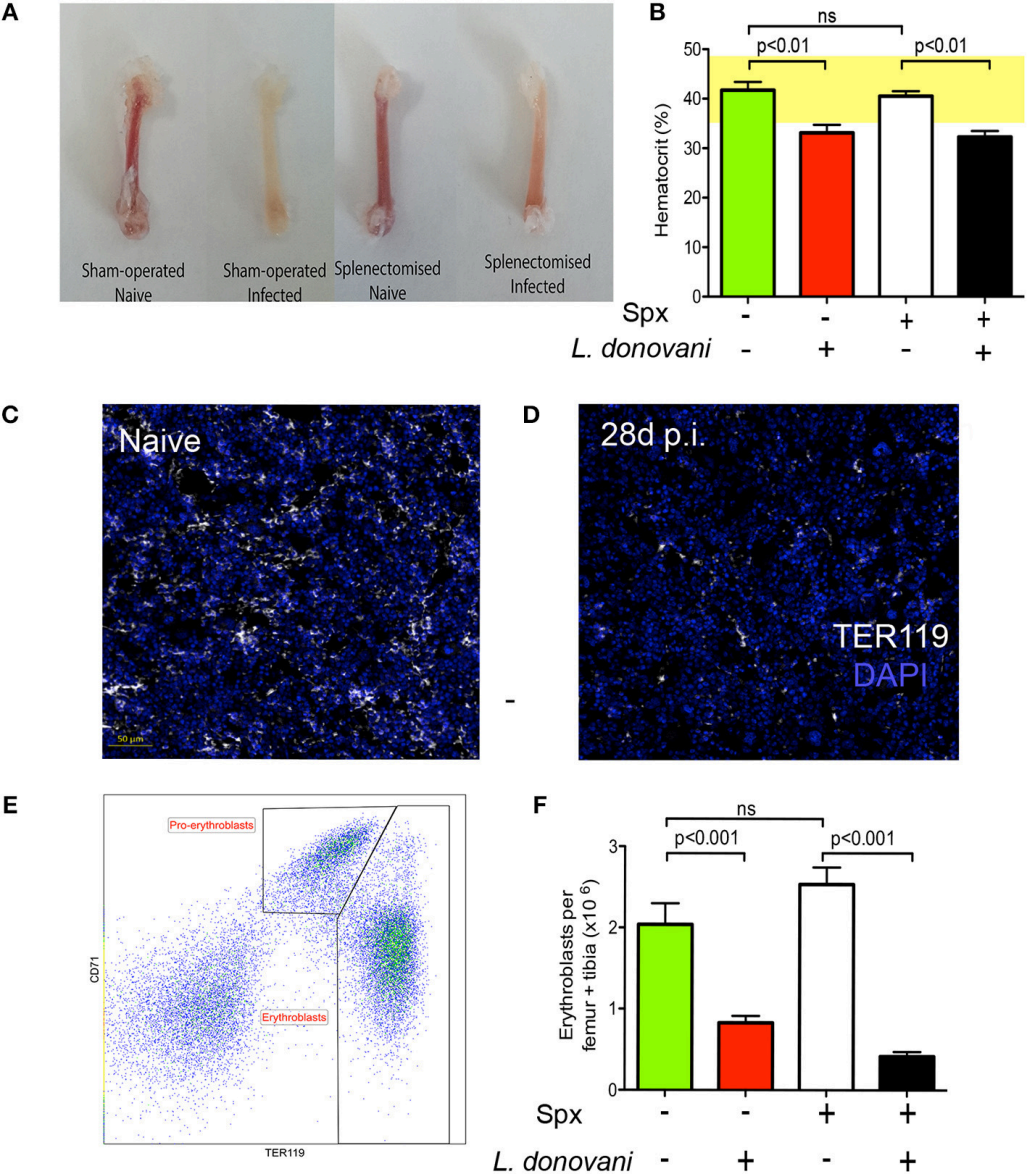
Medullary erythropoiesis is repressed during experimental visceral leishmaniasis. **(A)** Femurs isolated from *L. donovani*-infected mice and age-matched naïve mice. Representative from 30 mice per group from 10 independent experiment. **(B)** Hematocrit in naïve and infected mice with and without splenectomy (Sp_x_). **(C,D)** Confocal imaging of 5 μm-thick femoral sections from naïve **(C)** and infected **(D)** mice stained with DAPI (blue) and TER119 (white). Representative of six mice per group from two independent experiments. **(E)** Representative flow cytometry analysis of CD45^−^ BM cells from infected mouse using the erythroid surface markers CD71 (transferrin receptor) and TER-119. Pro-erythroblasts are CD45^−^ CD71^+^ TER119^low^ and erythroblasts are CD45^−^ CD71^−/+^ TER119^high^. **(F)** Absolute number of pro-erythroblasts per femur + tibia in sham operated and Sp_x_ mice. Mann Whitney test; *n* = 14 mice per group from four independent experiments. Data represent mean ± SEM. All experiments were performed 28 days post-infection.

To determine how anemia and medullary erythropoiesis were altered in the presence or absence of a spleen, we next compared the BM of splenectomized and sham-operated C57BL/6 mice. Decolouration of the femurs was observed in the presence and to a lesser extent in the absence of a spleen (Figure 2A). Likewise, hematocrit as a measure of anemia was significantly reduced independently of the presence or absence of a spleen (Figure 2B). We then stained femur sections with TER119. Nucleated TER119^+^ cells were clearly reduced in the BM of infected mice as determined by confocal microscopy (Figures 2C,D). In contrast to spleen, flow cytometry with CD71 and TER119 indicated that the number of pro-erythroblasts (CD71^+^TER119^low^ cells) in BM was similar between naïve and infected mice (0.32 ± 0.08 vs. 0.28 ± 0.06) whereas the number of erythroblasts (CD71^−/+^TER119^high^ cells) in infected mice was significantly reduced compared to the naïve mice (2.66 ± 0.16 vs. 0.55 ± 0.14; Figures 2E,F). A similar change in erythroblast number was also observed in mice splenectomized prior to infection. Prior to day 28 p.i, we observed no significant alteration in the frequency of BM erythroid precursors (Figure S2). Taken together with the data reported in Pinto et al. (28), showing that infection does not affect the absolute number or frequency of myeloid-erythroid progenitors (MEPs) in bone marrow, our data suggest that only the final stages of BM erythropoiesis are impaired in *L. donovani*-infected mice, and that this occurs independently of splenomegaly and splenic function.

### Myelogram of BM

To further characterize changes in cellularity of the BM, myeloid and erythroid cells were analyzed by differential counting (Table 2). Infected mice had an increased myeloid: erythroid ratio. Notably, infected mice had an increase in the index of myeloid maturation compared to naive mice, characterized by a high frequency of immature myeloid cells with a decrease in mature myeloid cells. A significant reduction of enucleated mature erythroid cells was also observed, suggesting disturbance in the maturation process and consistent with the anemia observed in blood. In contrast, the frequency of lymphocytes and macrophages was elevated. By morphology, alterations suggestive of dysplasia in the myeloid and erythroid series, including maturation asynchrony (nuclei: cytoplasm asynchrony), giant band cell, megalocyte, fragmented nuclei, binucleated cells and/or bilobed nuclei and atypical mitosis were all observed in infected mice. Other findings included emperipolesis and leuco-erythrophagocytosis (Figure S3).

**Table 2:** Comparative myelogram of naïve mice and mice infected with *L. donovani* for 28 days.

	**Naive**	**Infected**
Myeloid: Erythoid ratio	1.5 (1.3–2.0)	2.1 (1.7–2.8)^*^
Precursor myeloid: Mature myeloid	0.02 (0.01–0.03)	0.1 (0.04–0.19)^*^
Nucleated erythroid precursor: Nucleated erythroid mature	0.02 (0.01–0.03)	0.03 (0.02–0.05)
Precursor myeloid cells (%)	1.0 (0.6–1.1)	4.8 (2.6–6.0)^*^
Mature myeloid cells (%)	39.7 (35.5–42.5)	34.8 (31.0–38.1)^*^
Nucleated erythroid precursor cells (%)	0.6 (0.4–0.9)	0.6 (0.2–0.9)
Nucleated erythroid mature cells (%)	26.8 (19.4–30.6)	17.8 (11.8–21.1)^*^
Lymphocytes (%)	33.0 (26.4–37.4)	41.2 (35.7–47.2)^*^
Plasma cells (%)	0.4 (0.2–0.6)	0.6 (0.2–1.0)
Monocytes (%)	0.0 (0.0–0.2)	0.3 (0.0–0.7)
Macrophages (%)	0.0 (0.0–0.2)	0.0 (0.0–0.1)

Bold values are significant:

* *p < 0.05*.

### The Bone Marrow Microenvironment Is Altered During EVL

**Figure 3:**
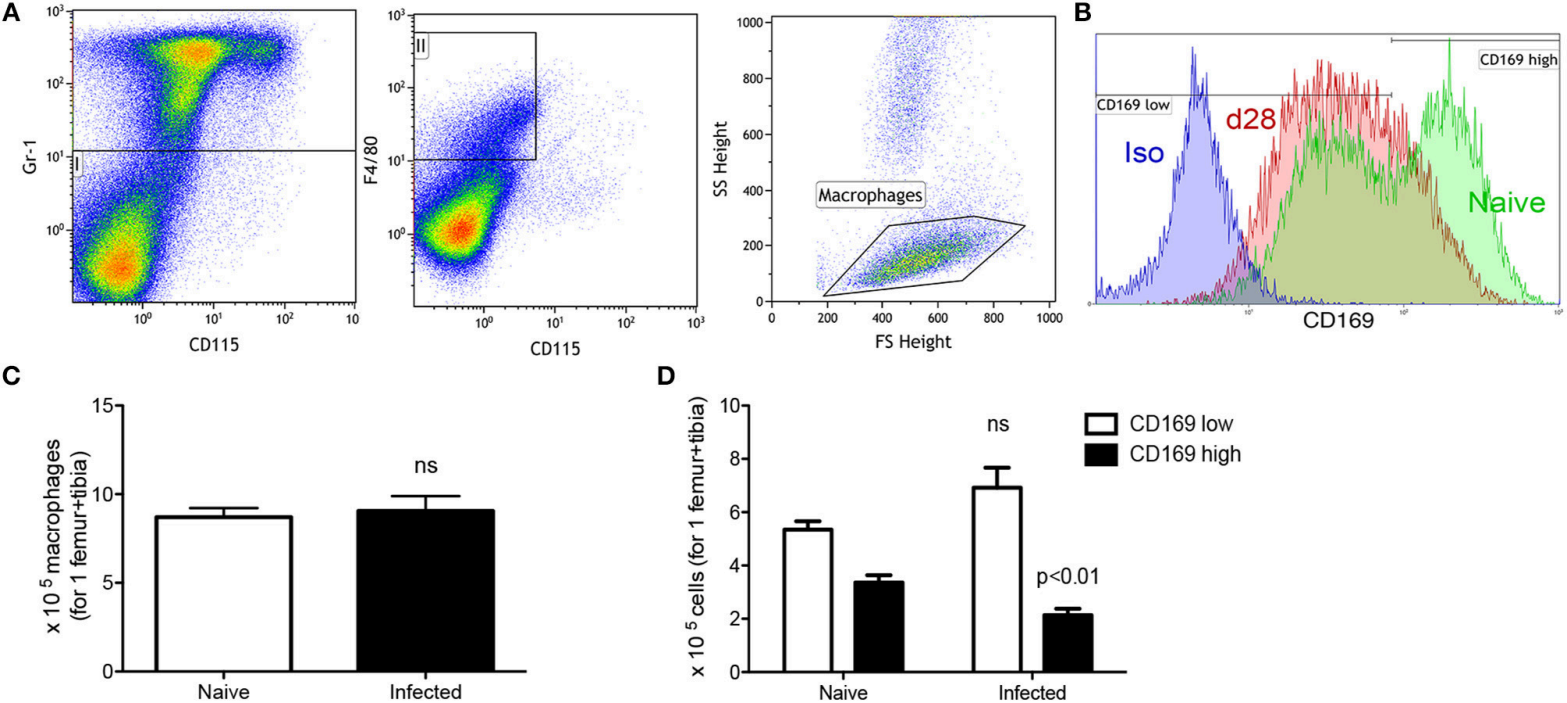
Infection with *L. donovani* reduces the number of CD169^+^ stromal macrophages in BM. **(A)** BM stromal macrophages were identified as Gr-1^−^ CD115^−^ F4/80^+^ SSC^low^ cells (9). **(B)** CD169 expression on BM macrophages of naïve (green) and infected (red) mice. Isotype control (blue) is representative of both naïve and infected mice. **(C)** Absolute number of macrophages per leg (1 femur + 1 tibia) according to the gating described in **(A,B)**. **(D)** Absolute number of CD169^low^ and CD169^high^ stromal macrophages based on gating in **(B)**. Absolute numbers were calculated from the frequencies multiplied by the total bone marrow cells isolated from each mouse. Data represent mean ± SEM. All experiments were performed 28 days after infection (unpaired *t*-test; *n* = 10 mice per group from two independent experiments).

To focus more specifically on cellular changes associated with erythropoiesis, we next examined two major components of the erythropoietic niche, stromal macrophages and CXCL12-abundant reticular (CAR) cells. CD169^+^ BM stromal macrophages have been reported by others to be important for supporting the later stages of erythropoiesis (7) and are identified as Gr-1^−^ CD115^−^ F4/80^+^ low side scatter (SSC^low^) cells (7) with surface expression of CD169 (Figures 3A,B). In naïve mice, CD169^low^ and CD169^high^ stromal macrophages could be clearly resolved (Figure 3B). Although the total number of Gr-1^−^ CD115^−^ F4/80^+^ SSC^low^ macrophages was similar between infected and naïve mice (Figure 3C), the ratio of CD169^low^: CD169^high^ populations was significantly altered. In naive mice, CD169^low^ macrophages accounted for 2.77 ± 0.59% of bone marrow cells or ~5 × 10^5^ cells per femur/tibia, whereas CD169^high^ stromal macrophages accounted for 1.70 ± 0.29% of total bone marrow cells (~3.5 × 10^5^ per femur/tibia). In contrast, in infected mice a clear population of CD169^high^ stromal cells was not apparent (Figure 3B), and numbers of cells gated as positive for CD169 expression was reduced to 2.14 × 10^5^ per femur/tibia (Figure 3D). These data suggest that either there is a loss of CD169 expression by BM stromal macrophages as a consequence of the environment created by infection, or that these cells are lost and replaced in equivalent numbers by other macrophages that lack CD169. The latter is consistent with the evidence provided above of enhanced BM myelopoiesis (Table 2).

**Figure 4:**
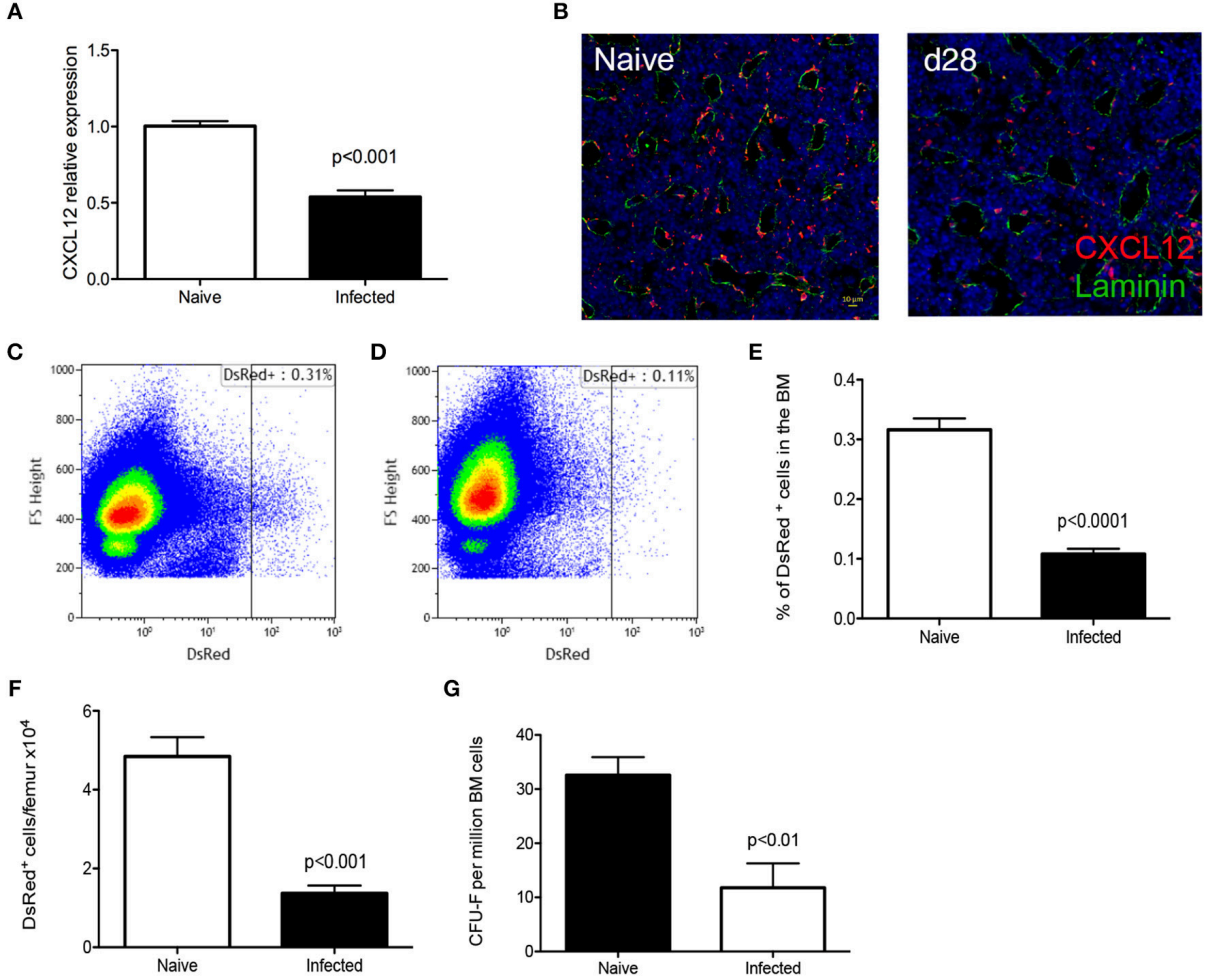
*L. donovani* infection causes a reduction in CXCL-12-expressing cells in the BM. **(A)** *Cxcl12* mRNA accumulation in BM of naïve and infected mice, determined by qRT-PCR. **(B)** Visualization of CXCL12-expressing cells using naïve and infected *Cxcl12*-DsRed reporter mice. Sectioned were co-stained for laminin (green) and counterstained with DAPI (blue). **(C,D)** Flow cytometry analysis of DsRed^+^ cells in naïve **(C)** and infected **(D)** *Cxcl12*-DsRed reporter mice. Dot plots show identical number of cells, gated on live single cells. **(E)** Frequency of DsRed^+^ cells. **(F)** Absolute number of DsRed^+^ cells per femur, calculated from the frequency of DsRed^+^ cells in **(E)** multiplied by the total bone marrow cell count (Mann Whitney test; Data from five naïve mice and nine infected mice from two independent experiments). **(G)** Number of CFU-F per million BM cells (Unpaired *t*-test; *n* = 7 mice per group from two independent experiments). Data represent mean ± SEM. All experiments were performed 28 days post-infection with *L. donovani*.

CD169^+^ stromal macrophages are known to interact with stromal reticular cells that produce CXCL12 (CAR cells) and that these are composed of mesenchymal stem and progenitor cells MSPCs (31). Therefore, we examined expression of CXCL12 at both protein and mRNA levels. RT-qPCR analysis of total BM cells from chronically infected C57BL/6 mice indicated a 50% reduction in *Cxcl12* mRNA accumulation compared to naïve mice (Figure 4A). We next used CXCL12 reporter mice to identify and quantitate CAR cells expressing this chemokine. By confocal microscopy, there was a clear reduction in the frequency of cells expressing CXCL12 in infected compared to naïve mice (Figure 4B). As the extensive ramifications of these cells made quantification difficult, we performed flow cytometry to validate these data (Figures 4C,D). In naïve B6.*Cxcl12*^DsRed^ mice, the frequency of CAR cells was 0.32 ± 0.02% of total bone marrow cells, corresponding to 4.84 ± 0.49 × 10^4^ cells per femur/tibia. In contrast, the frequency and absolute number of Ds-Red^+^ cells were reduced in infected mice (0.11 ± 0.01% and 1.36 ± 0.20 × 10^4^ cells per femur) (Figures 4E,F). Finally, to provide a functional confirmation of reduced numbers of CAR cells, we made use of the property of these cells to generate adherent fibroblastic colonies (CFU-F) *in vitro* (32). We found a reduction in the absolute number of CFU-F in the BM of infected mice (from 32.6 ± 3.4 CFU-F/1 × 10^6^ BM cells to 11.8 ± 4.5 CFU-F/1 × 10^6^ BM cells in naïve and infected mice, respectively; Figure 4G). Taken together, these results suggest that mice infected with *L. donovani* have reduced levels of stromal cell support for late-stage erythropoiesis in the BM.

### Bone Marrow Failure Is Linked to the Adaptive Immune Response

**Figure 5:**
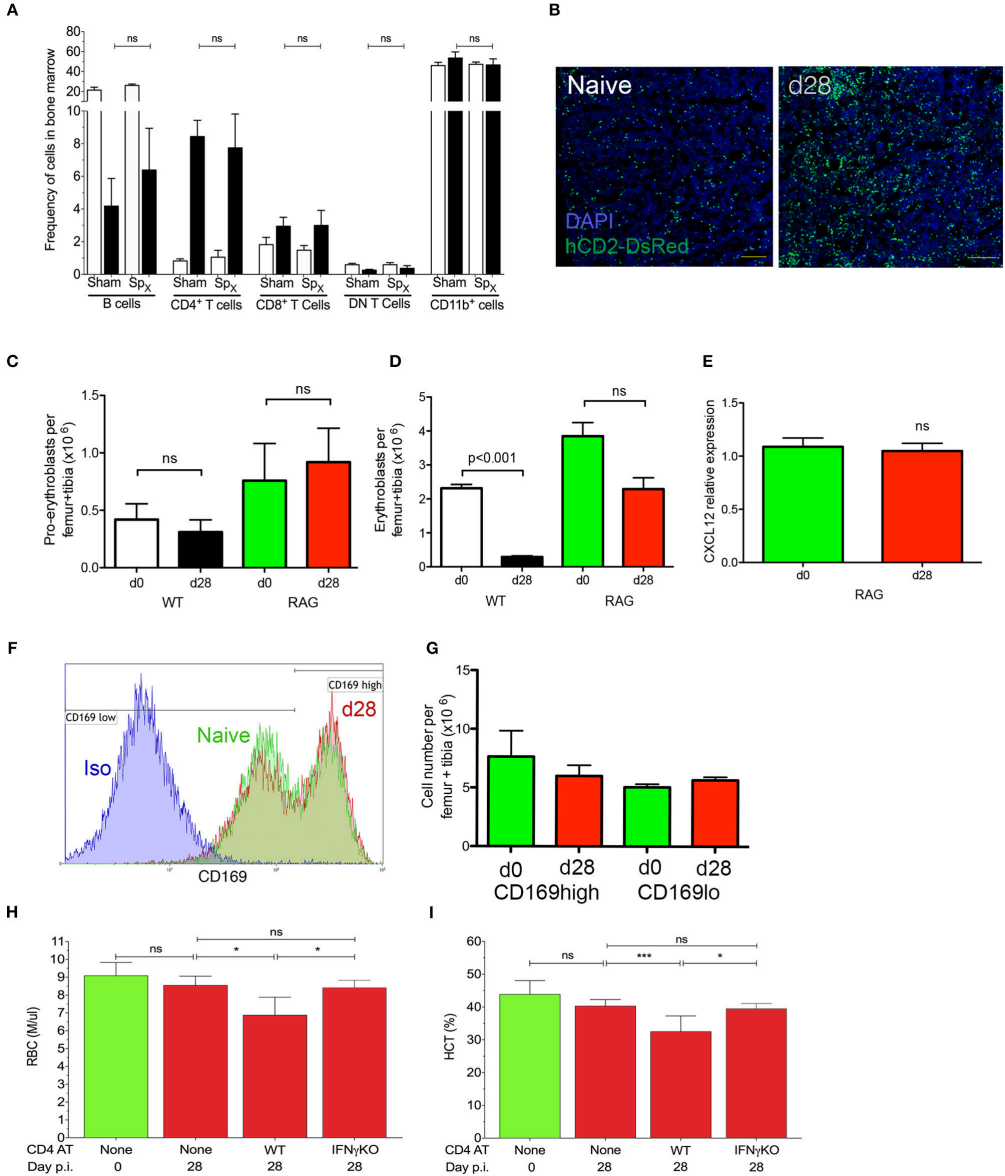
IFNγ-producing CD4^+^ T cells mediate repression of medullary erythropoiesis in experimental VL. **(A)** Frequency of leucocyte subsets accumulating in the BM of sham-operated or Sp_x_ naïve (open bars) and infected (black bars) mice. Data from one experiment (*n* = 5) mice per group; Mann-Whitney: not significant (ns). **(B)** T cell accumulation in BM visualized using hCD2-DsRed mice. Sectioned were counterstained with DAPI (blue). Femurs representative of 15 mice per group examined from three independent experiments. **(C,D)** Absolute numbers of pro-erythroblasts **(C)** and erythroblasts **(D)** in the BM of naïve and infected wild type C57BL/6 or B6.*Rag2*^−^^/−^ mice. Absolute numbers were calculated by multiplying frequencies by the total BM cell counts (One-way ANOVA with Turkey's multiple comparison test; *n* = 10 mice per group from two independent experiments). Data represent mean ± SEM. **(E)** *Cxcl12* mRNA accumulation in total BM cells from naïve and infected B6.*Rag2*^−/−^ mice. Intra-sample standardization was performed by normalization to HPRT and inter-sample standardization was done by normalization to the average expression of the naïve group (*n* = 8 wild-type mice per group, five naïve and seven infected from one experiment). **(F)** CD169 expression on BM macrophages of naïve (green) and infected (red) B6.*Rag2*^−/−^ mice. Isotype control (blue) is representative of both naïve and infected mice. **(G)** Absolute numbers of macrophages per leg (1 femur + 1 tibia), calculated from the frequencies multiplied by the total bone marrow cells isolated from each mouse (*n* = 3 naïve and four infected mice from one experiment). **(H,I)** Anemia, measured as RBC count **(H)** or hematocrit **(I)** in B6.*Rag2*^−/−^ mice receiving adoptive transfer of either IFNγ-sufficient (WT) or IFNγ-deficient (IFNγKO) CD4^+^ T cells [*n* = 4/5 per group; One-way Anova followed by Tukey's multiple comparisons test: not significant (ns), *p ≤ 0.05].

In addition to being a site of hematopoiesis, the BM is also a site of *L. donovani* infection (25, 28). To determine whether cell mediated immunity impacted on medullary erythropoiesis, we first assessed the number of lymphocytes in the BM of infected mice. As previously described (28), both CD4^+^ and CD8^+^ T cells were found to accumulate in the BM of infected mice, though an expansion in the frequency of CD4^+^ T cells represented the major change observed (Figure 5A, Figure S4). Accumulation of T cells was also confirmed by confocal microscopy of femur sections in B6.hCD2-GFP mice (Figure 5B). In contrast, we observed no change in the frequency of CD1b^+^ cells and a compensatory decrease in the frequency of B cells. Of note, similar changes were also observed in mice which had undergone splenectomy prior to infection, indicating that the spleen plays a limited role in the accumulation of bone marrow-homing T cells during infection (Figure 5A).

We next examined erythropoiesis in the BM of B6.*Rag2*^−/−^ mice by flow cytometry to determine whether adaptive immunity played a role in the suppression of medullary erythropoiesis. As in wild type mice, B6.*Rag2*^−/−^ mice infected with *L. donovani* had similar numbers of pro-erythroblasts as control uninfected mice (Figure 5C), despite significantly higher systemic parasite burden (Figure S5). In contrast, whereas wild type mice had significantly reduced numbers of erythroblasts, only a modest and not significant reduction in these cells was observed in infected B6*.Rag2*^−/−^ mice (Figure 5D). Similarly, B6.*Rag2*^−/−^ mice showed no reduction of *Cxcl12* mRNA accumulation after 4 weeks of infection compared to the ~50% reduction seen in wild-type mice (Figure 5E). In addition, there was no change in the expression of CD169^high^ on Gr-1^−^ CD115^−^ F4/80^+^ SSC^low^ bone marrow macrophages (Figure 5F), and the ratio of CD169^low^ and CD169^high^ bone marrow stromal macrophages was similar between the infected and naïve RAG2^−/−^ mice (Figure 5G).

Finally, we reconstituted B6.*Rag2*^−/−^ mice by adoptive transfer of CD4^+^ T cells prior to infection with *L. donovani*. B6.*Rag2*^−/−^ mice receiving CD4^+^ T cells displayed anemia similar to wild type immunocompetent mice, as measured by both erythrocyte count and hematocrit (Figures 5H,I). In contrast to these results obtained using adoptively transferred wild type CD4^+^ T cells, CD4^+^ T cells isolated from IFNγ-deficient B6.*Ifn*γ^−/−^ mice we unable to induce anemia (Figures 5H,I), despite equally efficient engraftment and activation (Figure S6). IFNγ KO T cells are defective compared to wild type CD4^+^ T cells in terms of controlling systemic parasite load (28). Collectively, these data support the conclusion that both the medullary changes in erythropoiesis and peripheral anemia seen in experimental VL arise as a consequence of CD4^+^ T cell activation and IFNγ production, independently of any potential contributions from splenomegaly.

## Discussion

Although evidence abounds that VL causes hematological alterations in humans, dogs and experimental model, such as hamsters, very little is known about the underlying mechanisms. In the present study, we show using an experimental murine model that CD4^+^ T cell-dependent adaptive immune responses to *L. donovani* underpin anemia through a pathway that involves repressed BM erythropoiesis consequent on alterations in the stromal microenvironment of the erythropoietic niche.

We show here that C57BL/6 mice chronically infected with *L. donovani* presented with a bi-cytopenia characterized by normocytic anemia and thrombocytopenia. These findings are consistent with the hematological data typically reported in human studies of VL, though indicate that in this strain of mice at least, there is no accompanying leucopenia. Anemia is often complex and multifactorial and it is likely that different models of disease may to a greater or lesser extent exemplify different underlying mechanisms. For example, multiple mechanisms have been proposed based on clinical observations for the profound anemia observed in human VL, including immune-mediated hemolysis (33) or splenic sequestration (10, 33, 34). In hamsters infected with *L. donovani*, anemia associated with lethal infection was correlated with increased apoptosis of erythroid progenitors and an increase of IFNγ in the BM and spleen (21). Our data in murine VL indicates that the spleen may have counteracting roles, on the one hand permitting enhanced erythrophagocytosis (21), but on the other serving as a site of extramedullary compensatory erythropoiesis. Indeed, it is likely that these events may balance each other, resulting in a mild anemia in intact mice that is subsequently unaltered by splenectomy. The fact that a mild anemia is present in infected mice independent of the presence or absence of a spleen, with dysplastic erythroid features, provides a convenient tool to allow exploration of pathological mechanisms operating within the BM microenvironment. Although we also observe thrombocytopenia in *L. donovani*-infected mice, the mechanisms regulating this process appear distinct from that controlling erythropoiesis and will be reported elsewhere.

Our analysis of the BM microenvironment that supports erythropoiesis has for the first time demonstrated that anemia in murine models of VL represents an aspect of CD4^+^ T cell mediated immunopathology. BM resident stromal macrophages, identified by the expression of the sialoadhesin CD169 (35), were reduced in number in infected mice. CD169^+^ stromal macrophages have been shown to be essential for stress erythropoiesis e.g., following chemically-induced anemia, but their depletion causes minimal disruption of physiological erythropoiesis. In these studies, there was no correlation between overt anemia and a reduction of erythroid progenitors in the BM (7). These data are in line with our observations, since in our model of EVL, chronic infection results only in a mild anemia despite a dramatic reduction of erythroid progenitors in the bone marrow as observed in myelogram and flow cytometry analysis. We have previously shown that *L. donovani* amastigotes readily parasitize CD169^+^ BM stromal macrophages during chronic infection and that infection of these cells directly supports an increase in their capacity to support myelopoiesis (26). Our current data extends these observations by indicating that the reduction of the number of CD169^+^ stromal macrophages is not a direct consequence of parasitism, as infected B6.*Rag2*^−/−^ mice have significantly increased parasite loads in the BM, yet show no changes in stromal macrophage number. Rather, our data suggest that loss of stromal macrophages is a further consequence of T cell dependent immune responses.

While CD169^+^ stromal macrophages were reduced in number, the total number of BM macrophages remained stable or increased during infection. It is unclear if loss of CD169^+^ stromal macrophages represents depletion or conversion to a different phenotype, for which specific lineage tracking studies would be required. STING-mediated activation of BM CD169^+^ macrophages has been shown to be essential to type I IFN production by plasmacytoid dendritic in a malaria mouse model (36), indicating that these cells are directly sensitive to infections. Similarly, dexamethasone treatment induces CD169 expression on the surface of human macrophages, promoting in the same time their erythropoiesis-supporting function (37). Hence, a stromal “CD169” phenotype can be acquired in differentiated macrophages and is responsive to inflammatory signals. Interestingly, dexamethasone is also an inhibitor of iNOS (38), thus suggesting a role for NO in EVL-induced anemia. Previously, CD169^+^ macrophages have been shown to be depleted by G-CSF administration (39). We have observed a consistent upregulation of circulating G-CSF in infected mice (data not shown) but to date our attempts to convincingly neutralize G-CSF *in vivo* have been unsuccessful. Hence, direct evidence is still needed to support a role for G-CSF in VL-induced anemia.

In hamsters and mice, infection with *L. donovani* causes an increase of erythroid burst forming units (BFU-E) from the bone marrow in colony formation assays (22, 25). These represent very early progenitors of erythroid cells, prior to the pro-erythroblast stage. In the current study, we show by flow cytometry that only later stages of erythroid differentiation, at or after the pro-erythroblast stage, are affected by infection. This is also reflected in differential counts of bone marrow cells, showing that nucleated mature erythroid cells were reduced in infected mice. Furthermore, conditional depletion of CD169^+^ cells in a mouse model did not alter the BFU-E content in the BM of mice (7). These data are collectively consistent with macrophage-dependent erythroblastic islands functioning to support erythropoiesis from the erythroblast stage onwards.

We also report that CXCL12-producing mesenchymal stromal cells are affected during VL. Infection led to a reduction of *Cxcl12* mRNA accumulation in the bone marrow, correlating with a reduction in the number of CXCL12-expressing cells. The main mechanism of G-CSF-induced down-regulation of CXCL12 is protease-dependent (40) but a more complex model including transcriptional regulation has also been reported. While down-regulation of CXCL12 is a potentially due to up-regulation of G-CSF, CD169 macrophages are also responsible for the retention of CAR cells in the bone marrow. It is likely that these mechanisms together factor into the loss of stromal support in the BM. In the case of *E coli*. infection, heightened levels of G-CSF led to a reduction in CXCL12 expression in the BM via Toll-like receptor and NOD1/2 signaling (3). This study did not however directly enumerate CXCL12-producing cells in BM, our observations here being the first reported instance of loss of these cells during infectious disease.

In summary, we have shown that IFNγ-producing CD4^+^ T cells contribute to anemia in a model of VL, via a mechanism that involves loss of both macrophages and mesenchymal stromal elements from the BM erythropoietic niche leading to dyserythropoiesis. Whether these effects are the result of direct IFNγ signaling on CD169^+^ macrophages and/or mesenchymal stromal cells, whether they reflect indirect effects of IFNγ on third party cells or whether they are the consequence of induced expression of one of the many IFN-responsive genes remains to be determined. We have also recently shown that CD4^+^ T cells producing both IFNγ and TNF accumulate in large numbers in the BM of infected mice, via a mechanism requiring CD4^+^ T cell-intrinsic TNF receptor signaling. These cells drive functional exhaustion within the long-term HSC compartment (28). Collectively, therefore, a picture emerges whereby CD4^+^ T cells play a pathogenic role in the BM that leads to BM failure with both short and long-term consequences for hematological health. These data provide an imperative for similar studies in humans, to determine whether CD4^+^ T cells likewise have a causative role in the hematological changes associated with VL or indeed other infections where BM accumulation of activated effector T cells occurs.

## Author Contributions

OP and FP: Study design, experimental work, data analysis, manuscript preparation; AP, GR, and NB: Experimental work, data analysis; IH and HG: Study design, manuscript preparation, researcher supervision; PK: Project oversight, study design, manuscript preparation, researcher supervision.

### Conflict of Interest Statement

The authors declare that the research was conducted in the absence of any commercial or financial relationships that could be construed as a potential conflict of interest.
