# Supplementary material for: CD4+ T Cells Alter the Stromal Microenvironment and Repress Medullary Erythropoiesis in Murine Visceral Leishmaniasis
Source: Front Immunol. 2018 Dec 18;9:2958. doi: 10.3389/fimmu.2018.02958 (PMC6305626; doi:10.3389/fimmu.2018.02958)
Supplement: Supplementary file 1 [file Data_Sheet_1.pdf]

$CD4^+$ T cells alter the stromal microenvironment and repress medullary erythropoiesis in murine visceral leishmaniasis. Preham et. al.

S1 Table. Distribution of infected mice according to normal values of haematological parameters.

	Reference interval	*Distribution relative to reference interval (%)*		
		**Under**	**Within**	**Above**
WBC (x10^3/ul)	2.40 - 15.73	0	100	0
NE (x10^3/ul)	0.37 - 5.03	0	100	0
LY (x10^3/ul)	1.96 - 9.01	7.69	92.31	0
MO (x10^3/ul)	0.05 - 0.92	0	100	0
EO (x10^3/ul)	0.01 - 0.71	0	92.31	7.69
BA (x10^3/ul)	0.00 - 0.26	0	100	0
RBC (x10^6/ul)	7.04 - 9.18	69.23	30.77	0
HB (g/dl)	8.00 - 11.19	30.77	69.23	0
HCT (%)	35.12 - 48.60	61.54	38.46	0
MCV (fl)	47.30 - 55.92	0	76.92	23.08
MCH (pg)	10.16 - 13.56	0	100	0
MCHC (g/dl)	18.08 - 28.00	0	100	0
PLT (x10^3/ul)	241.20 - 924.80	38.46	61.54	0
MPV (fl)	3.60 - 5.00	0	23.08	76.92

Supplementary Figures

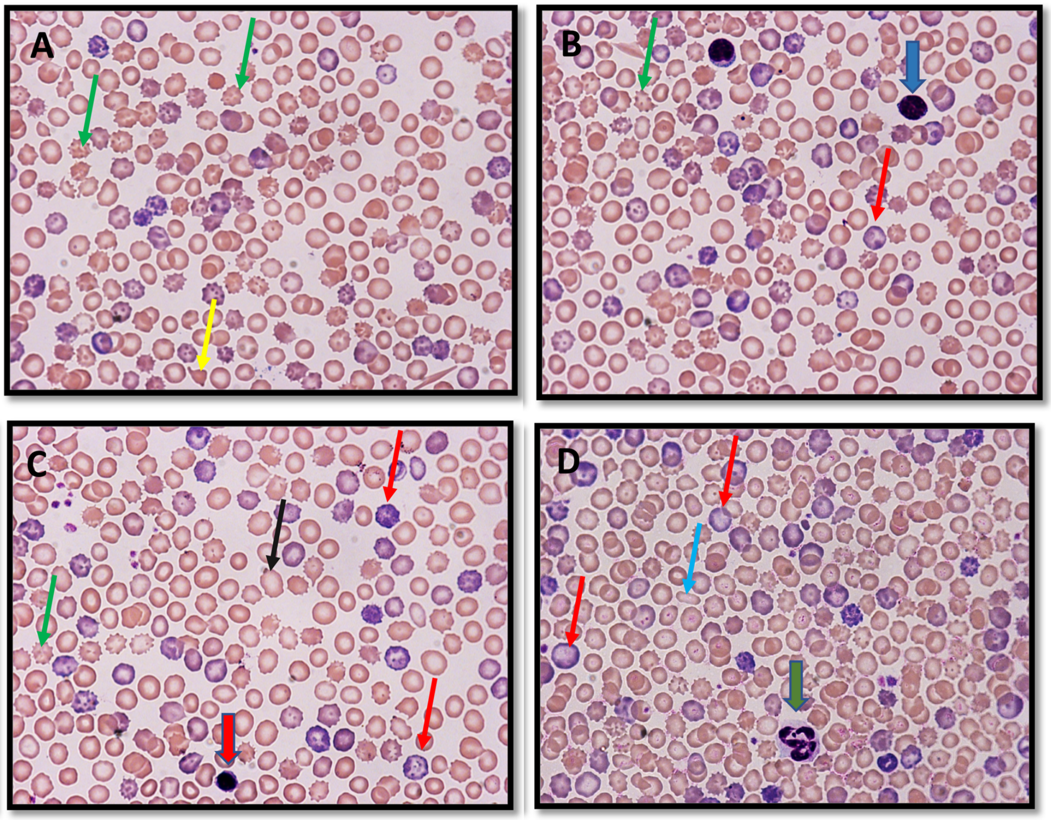

S1 Figure Aberrant red cell morphology following *L. donovani* infection

Representative images of M-G Giemsa-stained blood films from d28 *L. donovani*-infected mice. **A.** green thin arrow: acanthocytes; yellow thin arrow: schistocytes; **B.** red thin arrow: polychromatic red cells; blue large arrow: lymphocyte; **C.** red large arrow: nucleated red blood cell; black thin arrow: macrocyte; **D.** green large arrow: neutrophil. blue thin arrow: elliptocyte

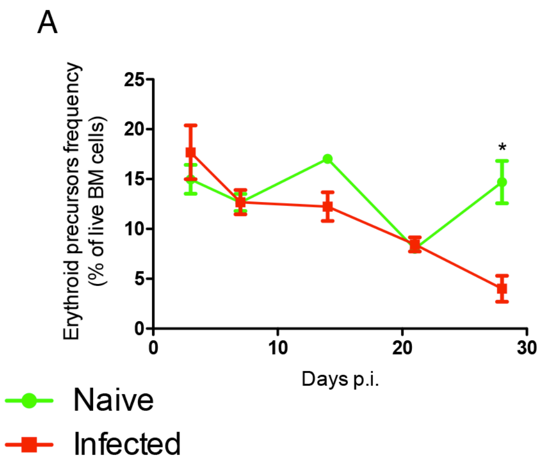

S2 Figure. Frequency of erythroid precursors in bone marrow.

Frequency of erythroid precursors (pro-erythroblasts and erythroblasts) in the bone marrow of naïve (green) and *L. donovani*-infected (red) B6 mice over time. Precursors were identified on the basis of TER119 and CD71 staining. Unpaired t-test; n=3 mice per group per timepoint).

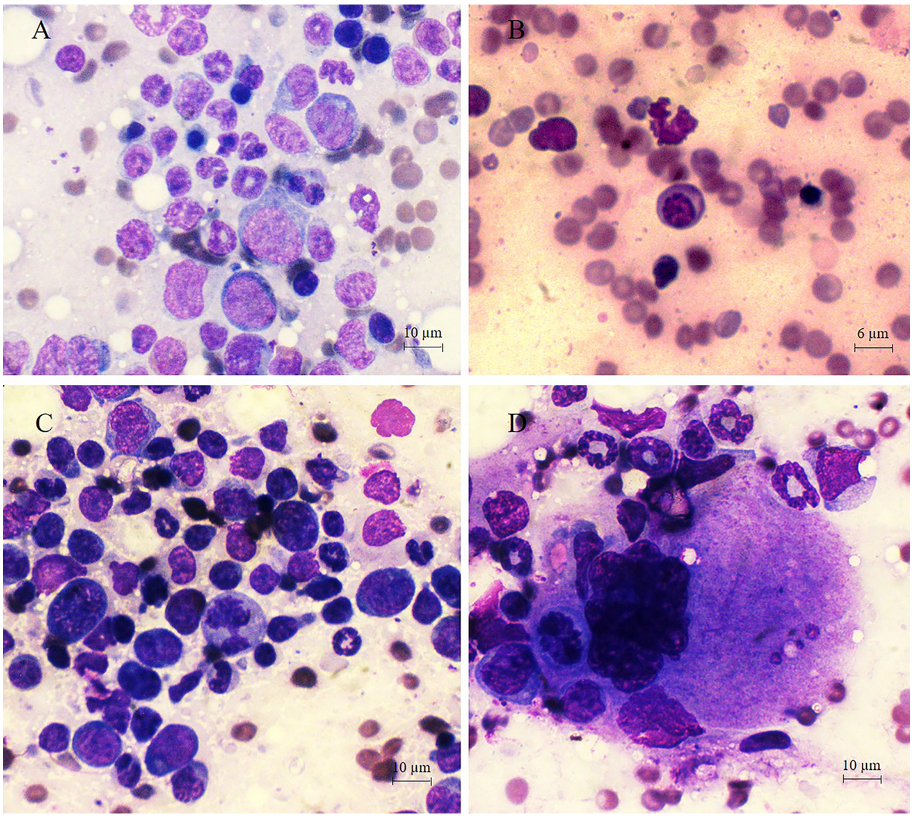

S3 Figure Myelogram of *L. donovani*-infected BM

BM samples were obtained by aspiration biopsy from iliac crest using 24 G needle attached to a 5mL disposable plastic syringe with 10% EDTA and smears were stained with May–Grünwald Giemsa and analyzed by optical microscopy (Zeiss, Germany) and images using Zen software (Carl Zeiss). A Binucleated erythroid cell. B Megalocyte. C. Atypical mitosis. D Emperipolesis.

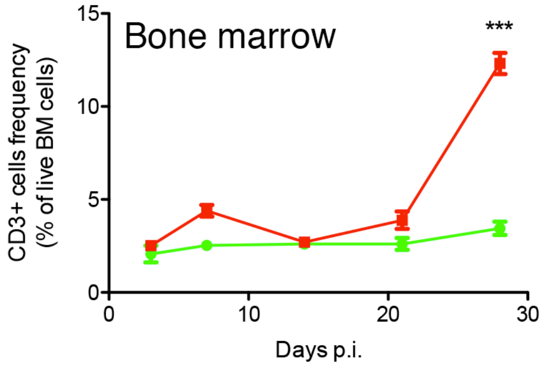

S4 Figure Frequency of T cells in bone marrow

Frequency of $CD3^+$ cells in the bone marrow of naïve (green) and *L. donovani*-infected (red) B6 mice over time. Unpaired t-test; n=3 mice per group per timepoint).

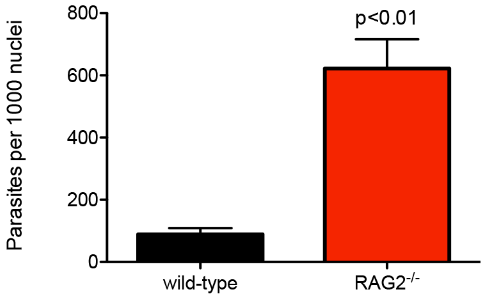

S5 Figure. Parasite load in *L. donovani* infected B6 and B6.*Rag2*$^{-/-}$ mice.

Parasites per 1000 nuclei in the spleen at d28 p.i.. Spleen impressions smears were made on glass slides and stained with Giemsa. Parasites and nuclei were counted microscopically. n=8 wild-type and 6 RAG2$^{-/-}$ mice from 2 independent experiments.

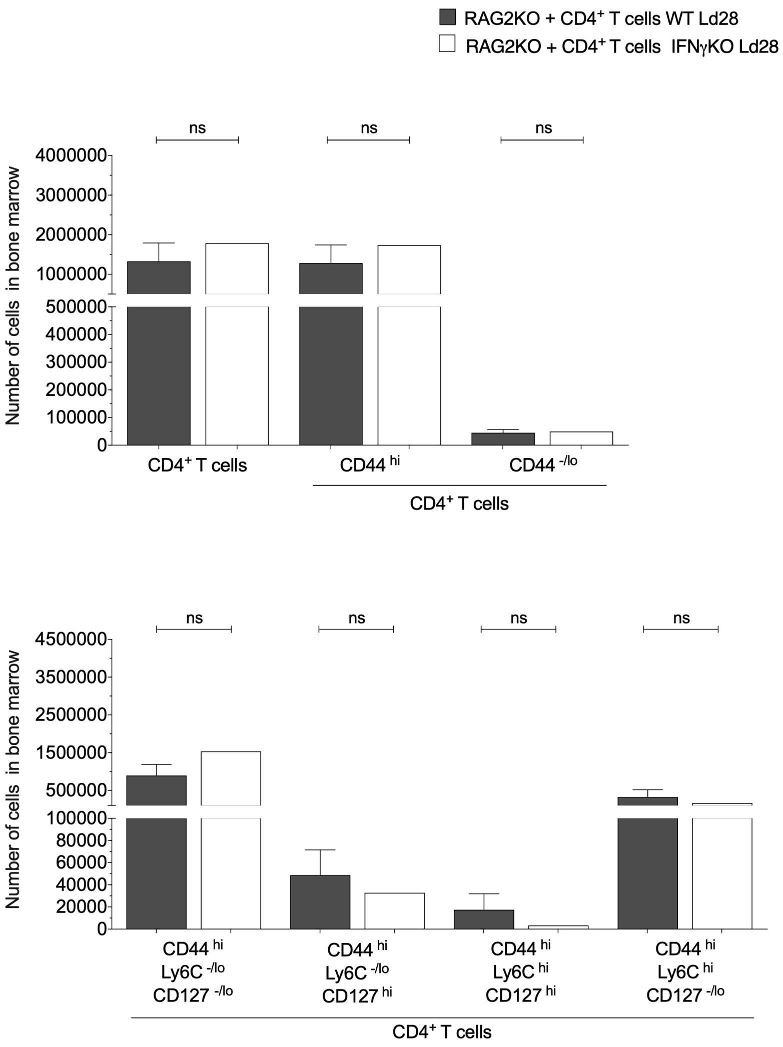

S6 Figure. Number and differentiation state of wild type and IFNγ KO CD4$^+$ T cells in RAG recipients.

Wild type (black bars) or IFNγ KO (open bars) CD4$^+$ T cells were transferred into RAG recipients prior to infection with *L. donovani*. At day 28 p.i., BM CD4$^+$ T cells were enumerated and characterized by flow cytometry. **A.** Number of total CD4$^+$ T cells and of CD4$^+$ T cells with CD44^{hi} and CD44^{lo} phenotype. **B.** Number of CD4$^+$ T cells expressing different expression patterns for Ly6C, CD44 and CD127. CD44^{hi}Ly6C$^{-/lo}$CD127$^{-/lo}$ are often regarded as classical effector cells. Two tibias and femurs were taken per mouse with n=4 mice receiving wild type T cells and n=5 mice receiving KO T cells.
